# Real-time virtual infection prevention and control assessments in skilled nursing homes, New York, March 2020—A pilot project

**DOI:** 10.1017/ice.2021.100

**Published:** 2021-03-19

**Authors:** Belinda E. Ostrowsky, Lauren M. Weil, R. Henry Olaisen, Rachel L. Stricof, Eleanor H. Adams, Marie I. Tsivitis, Antonella Eramo, Rosalie Giardina, Richard Erazo, Karen L. Southwick, Jane A. Greenko, Emily C. Lutterloh, Debra S. Blog, Crystal Green, Kimberly Carrasco, Rafael Fernandez, Snigdha Vallabhaneni, Monica Quinn, Sarah J. Kogut, Joy Bennett, David M. Chico, Martha Luzinas

**Affiliations:** 1 Division of Healthcare Quality Promotion, National Center for Emerging and Zoonotic Infectious Diseases, Centers for Disease Control and Prevention, Atlanta, Georgia; 2 Epidemic Intelligence Service, Division of Scientific Education and Professional Development, Center for Surveillance, Epidemiology, and Laboratory Services, Centers for Disease Control and Prevention, Atlanta, Georgia; 3 New York State Department of Health, Albany, New York; 4 New York State Department of Health (NYSDOH Metropolitan Regional Office), New Rochelle, New York; 5 University at Albany, SUNY, School of Public Health, Albany, New York; 6 New York State Department of Agriculture and Markets, Albany, New York

## Abstract

**Objective:**

To describe a pilot project infection prevention and control (IPC) assessment conducted in skilled nursing facilities (SNFs) in New York State (NYS) during a pivotal 2-week period when the region became the nation’s epicenter for coronavirus disease 2019 (COVID-19).

**Design:**

A telephone and video assessment of IPC measures in SNFs at high risk or experiencing COVID-19 activity.

**Participants:**

SNFs in 14 New York counties, including New York City.

**Intervention:**

A 3-component remote IPC assessment: (1) screening tool; (2) telephone IPC checklist; and (3) COVID-19 video IPC assessment (ie, “COVIDeo”).

**Results:**

In total, 92 SNFs completed the IPC screening tool and checklist: 52 (57%) were conducted as part COVID-19 investigations, and 40 (43%) were proactive prevention-based assessments. Among the 40 proactive assessments, 14 (35%) identified suspected or confirmed COVID-19 cases. COVIDeo was performed in 26 (28%) of 92 assessments and provided observations that other tools would have missed: personal protective equipment (PPE) that was not easily accessible, redundant, or improperly donned, doffed, or stored and specific challenges implementing IPC in specialty populations. The IPC assessments took ∼1 hour each and reached an estimated 4 times as many SNFs as on-site visits in a similar time frame.

**Conclusions:**

Remote IPC assessments by telephone and video were timely and feasible methods of assessing the extent to which IPC interventions had been implemented in a vulnerable setting and to disseminate real-time recommendations. Remote assessments are now being implemented across New York State and in various healthcare facility types. Similar methods have been adapted nationally by the Centers for Disease Control and Prevention.

On March 11, 2020, The World Health Organization declared the spread of severe acute respiratory coronavirus virus 2 (SARS-CoV-2), the novel coronavirus that causes coronavirus disease 19 (COVID-19), a pandemic.^1^ Around this time, the New York State Department of Health (NYSDOH) Metropolitan Area Regional Office (MARO) began receiving reports of suspected COVID-19 cases in skilled nursing facilities (SNFs). Residents of SNFs are typically older adults with underlying comorbidities, a population that has been identified as vulnerable to severe COVID-19 outcomes.^2–4^ In response, the NYSDOH issued a health advisory with infection prevention and control (IPC) directives for all SNFs and adult care facilities (ACFs) on March 13, 2020.^5^ These included preventing the introduction of SARS-CoV-2 in facilities by immediately implementing visitor restrictions, requiring all staff to wear face masks within 2 m (6 feet) of residents, and conducting temperature checks and symptom screening for everyone entering the facility. Additional IPC and healthcare guidelines were specified for facilities with residents having suspected or confirmed COVID-19. As the number of facilities impacted by COVID-19 increased, a more scalable solution was needed to develop communication channels between the NYSDOH and SNFs to provide guidance on evolving healthcare IPC recommendations and to supplement traditional on-site infection control assessments.

The NYSDOH and Centers for Disease Control and Prevention (CDC) field team developed a targeted COVID-19 IPC assessment for SNFs that was pilot tested in March 2020. The assessment included screening for suspected or confirmed COVID-19 cases in residents and staff, performing a telephone IPC checklist assessment, and conducting a COVID-19 video IPC assessment (COVIDeo). The pilot project had the following objectives: (1) establish situational awareness for public health and SNFs, (2) create a scalable approach to cover a wide geographical area during a short period of time, (3) assess the implementation of COVID-19 healthcare IPC recommendations, and (4) provide SNFs real-time quality improvement recommendations to address identified IPC gaps. This report describes the pilot project conducted in New York over a pivotal 2-week period when the area was becoming the nation’s epicenter for COVID-19.

## Methods

### Facilities

Licensed skilled nursing facilities (SNFs) in the 14-county greater metropolitan area, including New York City’s 5 boroughs (The Bronx, Manhattan, Queens, Brooklyn, and Staten Island), Dutchess, Nassau, Orange, Putnam, Rockland, Suffolk, Sullivan, Ulster, and Westchester counties.

The IPC tools were initially designed for proactive prevention-based assessments and were then adapted for response-based assessments. Proactive assessments were prioritized for SNFs near hospitals reporting COVID-19 cases, in new geographical areas, or in areas with widespread community transmission. Response-based assessments were prioritized for facilities reporting known COVID-19–positive residents or staff, deaths in COVID-19–positive patients, or clusters of influenza-like illness through several passive surveillance mechanisms.

### Personnel

In total, 10 public health epidemiologists with infection prevention expertise were involved in the development and/or use of the IPC assessment tools.

### Intervention approach

The IPC assessment was designed to facilitate a structured 2-way discussion of IPC recommendations with SNF administrators, directors of nursing, or infection preventionists. All assessments were nonregulatory.

### Screening tool

A screening tool was developed for situational awareness for public health and SNFs and included the facility census, facility layout, population served (ie, ventilator/tracheostomy, dialysis, dementia, and rehabilitation units or services), number of residents or staff with suspected or confirmed COVID-19 or with other intercurrent respiratory illnesses, number of floors or units affected, hospitalizations, and staff absenteeism (Supplementary Form 1 online).

### IPC checklist

The telephone checklist captured facilities’ self-reported assessment of the implementation of COVID-19 IPC recommendations (Supplementary Form 2 online). The checklist was adapted from the CDC’s “Preparing for COVID-19 in Nursing Homes”^6^ and “Coronavirus Disease 2019 (COVID-19) Preparedness Checklist for Nursing Homes and other Long-Term Care Settings” (original tool).^7^ In addition, it included the NYSDOH health advisory IPC directives from March 13, 2020.^5^

### COVIDeo

The COVIDeo tool provided direct observation of IPC recommendations implemented by facilities (Supplementary Form 3 online). These virtual visits were conducted using different video chat applications. The COVIDeo began outside the entrance to the facility, proceeded to the temperature and symptom screening station, moved to the lobby area, and then to a floor with residents on transmission-based precautions for COVID-19 or other reasons if there were no COVID-19 cases. Observations were made in common areas including dining rooms, activity rooms, hallways, and nurses’ stations. Environmental services (EVS) personnel were interviewed, and their carts were examined for disinfectants with Environmental Protection Agency (EPA) registration numbers.^8^ Where appropriate, real-time suggestions for identified IPC gaps or challenges were discussed.

To the extent possible, residents were not observed during COVIDeo visits. Video recording was not used. The video-based assessment process was undertaken as an emergency public health activity and therefore did not undergo review by an institutional review board per the NYSDOH.

### Data collection and aggregation

The screening tool was used to capture SNF demographics and number of facilities with suspected or confirmed COVID-19 cases; means, median, and ranges were calculated when appropriate. The number and percentage of SNFs reporting the implementation of 25 infection prevention elements were captured using the IPC checklist. For the COVIDeos, 6 key domains composed of 2–4 IPC elements were assessed. The number and percentage of SNFs that implemented the domains based on visual observation or elicited from the discussion were tallied. Qualitative themes from each of the 3 tools were summarized.

## Results

During March 15–28, 2020, IPC assessments were conducted in 92 SNFs with a median bed capacity of 200 (range, 150–266 beds): 16 (17%) provided care for mechanically ventilated residents, 12 (13%) had designated memory care units, and 8 (9%) had collocated hemodialysis units. For all 92 SNFs, the screening tool for potential COVID-19 activity and review of the IPC checklist were performed, taking 35–45 minutes per facility. COVIDeo was used in 26 (28%) of 92 SNFs that agreed to have a virtual assessment, which required 10–40 minutes depending on findings and discussion generated.

Of the 92 SNFs, 52 (57%) had reported COVID-19 cases, and 40 (43%) were conducted as proactive prevention assessments. Suspected or confirmed COVID-19 cases were identified in 14 (35%) of these 40 proactive assessments. For additional context, during the pilot project, the NYSDOH staff in MARO investigated a total 209 SNFs reporting suspected or confirmed COVID-19 cases in residents or staff. Of those, 21 traditional on-site visits were made to 12 (6%) of 209 affected facilities.

Based on the telephone checklist, facilities were able to universally implement visitor restrictions, and health checks at entrances (100%). Almost all SNFs implemented universal masking of staff with either an N95 respirator or a face mask (96%). The IPC implementation challenges included adequate access on units to alcohol based hand rub (ABHR) (71%), avoidance of floating staff between units (72%), informative signage specifying transmission-based precautions outside resident rooms (74%), active monitoring of all residents on COVID-19 affected units (85%), and access to necessary personal protective equipment (PPE) (87%) (Table 1).

**Table 1.** Number of Skilled Nursing Facilities Implementing COVID-19 IPC Recommendations Using the Telephone IPC Assessment Checklist

IPC Elements Assessed	No. (%)
**Facility restrictions and health checks**	
Implemented state required suspension of all visitation, expect essential visitors	92/92 (100)
Facility performing active health checks including temperature monitoring for everyone entering the building	92/92 (100)
All staff performing health checks are wearing face masks	92/92 (100)
Facility has cancelled group activities and communal dining	87/92 (95)
Signs posted on front of the facility advising no visitors	89/92 (97)
Essential visitors are required to wear a face mask and remain in the resident’s room	92/92 (100)
Staff with symptoms or temperature ≥37.8°C (100.0°F) are sent home	90/92 (98)
**Personal protective equipment (PPE)**	
Universal masking throughout the facility with a N95 or face mask	88/92 (96)
ABHR are easily accessible throughout the facility	65/92 (71)
Staff have PPE to implement contact and droplet precautions	80/92 (87)
Necessary PPE is available immediately outside resident rooms	81/92 (88)
Signs posted immediately outside resident rooms indicate appropriate transmission-based precautions and the required PPE	68/92 (74)
Trash disposal bins should be positioned near the exit of resident rooms	81/92 (88)
Ensure EPA-registered hospital grade disinfectants are available for frequent cleaning of high-touch surfaces and shared resident equipment	83/91 (91)
**Identification and management of ill residents in the facilities**	
A process is in place to identify and manage all residents with symptoms of respiratory infections daily	88/92 (96)
Residents wear a face mask when HCP or other care providers enter their rooms, unless such is not tolerable	77/92 (84)
Implement active monitoring of all residents on affected units once per shift. Monitoring must include symptom checks, vitals, lung auscultation, and pulse oximetry	78/92 (85)
All residents in affected units remain in their rooms as feasible	86/92 (93)
Do not float staff between units	66/92 (72)
**Occupational health**	
The facility instructs staff to regularly monitor themselves for fever and symptoms of respiratory infections, as part of routine practice	87/92 (95)
Facility is monitoring staff absenteeism for increased numbers and assessing the reasons	89/92 (97)
**Communications**	
Communication of interfacility transfer for residents with respiratory symptoms/suspect COVID-19; notify EMS transfer personnel and receiving facilities.	88/92 (96)
Proactively notify all resident family members about COVID-19 activity within the facility and measures implemented	88/92 (96)
**Surge capacity**	
Estimates should be made and frequent reassessments of the quantities of essential resident care materials and equipment and personal protective equipment.	82/92 (89)
A plan has been developed to address likely supply shortages (eg, personal protective equipment), including strategies for using normal and alternative channels for procuring needed resources.	84/92 (91)

Note. IPC, infection prevention and control; ABHR, alcohol-based hand rub; EPA, Environmental Protection Agency; EMS, emergency medical services.

COVIDeo visits identified additional IPC gaps and, in some cases, greater challenges than those reported on the checklist. For example, only 38% of facilities were observed to have accessible ABHR; 68% had informative signage specifying the type of transmission-based precautions; and 69% had easily accessible PPE outside resident rooms (Table 2).

**Table 2.** Number of Skilled Nursing Facilities Implementing COVID-19 IPC Recommendations Observed Using COVIDeo Assessment

IPC Elements Assessed	No. (%)	Meaningful Observations	Immediate Recommendations	Long-Term Recommendations
Facility implemented visitor restrictions	26/26 (100)	• Restrictions were quickly and efficiently put into place.	N/A	• Use as a model for the future to limit visitation during seasons of high influenza activity.
		• Facilities shared novel education/messaging to limit visitation and to explain COVID-19 concerns to patients/families.		
Facility implemented active health checks for persons entering the building (temperature check and symptom screening)	26/26 (100)	• Some facilities had excellent screening programs including a review of symptoms by healthcare personnel (HCP).	• The symptom screen should include a list of specific symptoms and be reviewed when any person checks a symptom or temperature exceeds threshold.	• Use as a model for the future to routinely discourage HCP from working while ill. Systems should be developed to track and screen sick calls from HCP, and sick leave policies should be strengthened.
		• In at least one facility, the symptom screen was limited to a check box stating, “feeling well.”		
Alcohol-based hand rub (ABHR) immediately available at entrance to resident rooms	10/26 (38)	• Although most facilities had some ABHR, dispensers were often sparsely placed at the entrance to a corridor, in the hallway, or at the nurse’s station.	• At a minimum, ABHR dispensers should be placed at entrance to facility, by elevators, at entrance to suspected or confirmed COVID-19 resident rooms, on medication carts and throughout hallways.	• ABHR dispensers should be placed in all common areas and at the entrance to all resident rooms.
		• Even when not available immediately inside or outside a resident room, ABHR was not available on the isolation cart/caddie. Hand hygiene access was limited to the residents’ bathrooms.		
Specific transmission-based signage outside resident rooms with required personal protective equipment (PPE) listed	17/25 (68)	• Signage was often limited to “Go See Nurse.”	• Best practice is for signage to depict or list the explicit PPE to be used to care for a resident on transmission-based precautions.	• Laminate all signs to ensure they can be cleaned, disinfected and reused.
		• Some signs were so complex with step-by-step directions and details for use that we could not determine what specific PPE was being recommended.		
PPE accessible at entrance to suspected or confirmed COVID-19 resident rooms	18/26 (69)	*PPE accessibility:*	• All necessary PPE should be readily accessible at the entrance to all resident rooms with suspected or confirmed COVID-19.	• All necessary PPE should be readily accessible at the entrance to rooms for residents on transmission-based precautions.
		• PPE/isolation carts were often empty or insufficiently stocked.		
		• Some facilities had many residents on precautions and ran out of PPE/isolation carts.		
			• Given current shortages, staff that have been provided PPE for extended use or reuse should have been provided instructions and training on proper donning and doffing, handling and cleaning and disinfection between use, if possible. This should include just-in-time training and retraining if new PPE products are introduced (ie, new masks, gowns).	
		• Eye protection was often not available due to shortages.		
		• Due to PPE shortages, some facilities resorted to limiting access to PPE (locked, in supervisors’ offices, etc). This resulted in limited access to necessary PPE.		
		• Severe PPE shortages resulted in extended use, reuse, or obtaining different brands of PPE or nonmedical products (rain ponchos, cloth masks, etc).		
			• Facilities should discuss PPE conservation strategies, review and simulate resident and nonresident care activity to prioritize and identify PPE needed.	
PPE disposal bins were available immediately inside the resident room	25/26 (96)	• PPE disposal was limited to the bathroom or resident’s waste container and was unable to safely contain the amount of waste generated.	• Waste containers for removal of PPE in doffing areas should be provided, with ABHR and disinfecting wipes in immediate proximity.	• Long-term processes for disposal should revert to usual processes (ie, for contact precautions before exit of room).
Use of EPA-registered disinfectant for SARS-CoV-2 and system for environmental and equipment disinfection	25/26 (96)	• When observed, some EVS personnel were spraying a cloth with disinfectant and wiping the surface. The “wet” time was 1–2 s instead of required contact time (1–10 min depending upon product).	• Ensure EPA-registered disinfectants are used at the appropriate concentration and for the required contact time for SARS-CoV-2.	• EPA-registered disinfectant wipes should be placed on isolation carts or caddies at the entrance to all resident rooms on transmission-based precautions as appropriate for specific infectious agent (eg, SARS-CoV-2).
		• Some facilities were not measuring to ensure appropriate dilution. We observed both over and under dilution (eg, 1:3 bleach solution).	• If available, EPA-registered disinfectant wipes for SARS-CoV-2 should be placed on isolation carts or caddies.	
		• Disinfection products were not near rolling/shared equipment, or a system for disinfection was not in place.	• A system should be in place to clean and disinfect common surfaces (PPE carts/nursing station) and medical equipment (eg, portable pulse oximetry).	
		• Common areas such as nursing stations had clutter.		
**General IPC Issues**				
PPE related use issues	Not assessed^a^	*PPE being used without proper training:*	N/A	• Assess the need and feasibility for a respiratory protection program with medical clearance and fit testing for appropriate HCP.
		• N95s were frequently being used in the absence of a respiratory protection program (without medical clearance or fit testing).		
		*PPE donned incorrectly or being used in redundant fashion:*		
		• HCP were seen wearing N95s over face masks, therefore face seal leakage could not be established.		
		• Face shields were being used over goggles, wasting limited resources.		
		• Face masks, especially cloth masks, resulted in frequent handling near eyes, nose, and mouth.		
		• Cloth face masks were seen with medical masks tucked in. They required frequent handling to remain in place.		
		*Improper PPE storage:*		
		• Cone-shaped respirators were stored folded in pockets, which could lead to contamination and impair facial fit characteristics.		
		• Gowns were reused after storing next to or on top of one another which could result in contamination.		
Congregate activities and variations for special populations	Not assessed^a^	• Although activity rooms were closed, residents were frequently seen in hallways, many without masks. Even when masks were seen on residents, they had often fallen to the neck.	• Identify modifications to limit transmission in populations where compliance with usual practices may be difficult (ie, dementia ward) such as treating the entire unit as potentially exposed, breaking the larger unit into smaller groups to limit exposures, limiting floating of staff, encouraging supervised hand hygiene and cough etiquette as able.	• Anticipate units or populations where IPC practices may be difficult and establish accommodations.
		• Residents with dementia or mental health issues posed a challenge for social distancing, due to inability to comply with infection control measures.		

Note. EVS, environmental services; HCP, healthcare personnel.

a General IPC issues were commonly observed, but not quantitively evaluated on the initial COVIDeo assessment.

Observations made using COVIDeo that could not be captured through the telephone checklist included errors in use and storage of PPE, limited knowledge in disinfecting surfaces, and difficulties in implementing resident movement restrictions (Table 2). Common themes included empty or insufficiently stocked isolation carts, healthcare personnel wearing PPE incorrectly or using PPE in a redundant fashion, PPE being improperly stored, and limited access to safely doff PPE. For example, in some areas, disposal bins were not located next to hand hygiene stations. Although 96% of facilities had EPA-registered “List N” products available, knowledge on use of healthcare-grade environmental surface disinfecting agents was limited. Residents were frequently seen in hallways, often without masks. The level of detail observed during COVIDeo assessments captured infection risks and allowed NYSDOH staff to deliver real-time quality improvement recommendations both immediately and for the long-term (Table 2).

## Discussion

The pilot project described in this report details remote IPC efforts implemented in SNFs when New York was becoming the epicenter of the COVID-19 pandemic^9^ and additional tools were needed to complement traditional public health investigation activities, such as on-site visits. The remote assessment tool established outreach with 92 SNFs and provided a structured method to deliver IPC education and outreach early in the pandemic when the understanding of the pathogen was limited, COVID-19 healthcare infection control guidelines were evolving rapidly, and the number of facilities impacted was increasing daily. To illustrate this point, within 10 days after initiation of this project, 89% of the 92 facilities had reported suspected or confirmed cases.

Initially intended for proactive prevention, the screening tool became a sentinel surveillance instrument as discussions about subtle presentations of COVID-19 in residents led to the recognition that many more SNFs were likely affected than had been realized previously. Our initial calls often fostered relationships that enabled discussions in the ensuing weeks as cases were identified at these facilities.

The telephone checklist provided a structured conversation for SNFs to discuss IPC challenges, and it provided a platform for targeted education and guidance on topics that were difficult to observe. Themes identified as challenges that were not easily observable included limiting staff floating between units and implementing the active monitoring of residents on COVID-19–affected units. Immediate suggestions to overcome these challenges were discussed, such as recommendations to cohort staff providing care to COVID-19–suspected or –confirmed residents and implementing vital checks, lung auscultation, and pulse oximetry for residents once per shift.

The COVIDeo component was a novel element using standard smartphone technology that allowed for real-time assessment by IPC subject-matter experts. Unlike the self-reporting captured during the telephone checklist, the observation of IPC practice provided opportunities for public health to recommend concrete suggestions for mitigating observed IPC gaps. COVIDeo provided several extra benefits including improved communication with SNFs through face-to-face interaction, more objective observations of IPC practices, and targeted quality improvement recommendations to address identified gaps in real-time in the actual setting of care. Although not formally documented, recommendations were shared in almost every COVIDeo that led to immediate improvements.

By eliminating commuting time, COVIDeo allowed for an estimated 4 times as many facilities to be assessed compared to on-site assessment while providing the virtual presence of public health epidemiologists. In addition, virtual visits removed additional exposure to the facility staff and residents. COVIDeos were well accepted, but they were conducted in fewer than one-third of facilities. There are several possible explanations for this; for example, the pilot project took place early in the COVID-19 pandemic when video technology was not as commonly used and facilities often lacked easy access to phone or computers video applications. Facility administrators and infection preventionists were experiencing heavy workloads and competing interests limiting available time. Additionally, virtual IPC assessment was an unfamiliar method, and administrators may have been skeptical at first even though they were nonregulatory. Public health epidemiologists were also gaining comfort with the tool and may not have consistently offered video assessments.

COVIDeos complemented traditional prevention or response efforts and were occasionally bundled with site visits where remote assistance was leveraged to provide the first assessment(s). In other circumstances, COVIDeos were used following on-site visits to demonstrate that basic recommendations had been adopted. Although some observed IPC challenges extended beyond the implementation of COVID-19–specific recommendations such as limited availability of ABHR in facilities. The COVIDeo visits addressed issues unique to COVID-19 that evolved during the response. These issues include helping facilities handle PPE shortages by educating staff on PPE optimization strategies,^10^ assuring proper donning, doffing, and reprocessing of PPE, and management of staff fears and misconceptions regarding COVID-19.

The NYSDOH used these IPC assessment tools in other regions of the state that were geographically remote from the epicenter (ie, had fewer recognized COVID-19 cases) to disseminate messages and expand reach. During March 25–April 1, 2020, IPC assessments were used for prevention-based assessment and situational awareness in 51 SNFs across 15 counties. Observations from these assessments were similar to those described during the pilot project.

This study had several limitations. First, interrater reliability was not measured. The infection prevention and control knowledge of the health department epidemiologists conducting the assessments could have biased the results of the telephone checklist and COVIDeo because skill level may have influenced how the elements were assessed. Second, although results obtained in SNFs in the greater New York metropolitan area are highly representative of this locale, they may not be generalizable to other areas. Third, the impact of the assessments was not systematically evaluated. Outcome measures for assessments were not feasible due to limited resources in the face of emergency. During this time, testing was not widely available and multiple interventions were implemented at once. Fourth, the entire tool kit took ∼1 hour to complete, which limited the number of facilities 1 investigator could reach per day. Also, the facilities that agreed to the COVIDeo assessments might have differed in their IPC implementation practices from those that only participated in the telephone checklist.

Although the tool did not fully replicate the benefits of on-site assessments, this pilot project established a platform with which to assess the current situation, the capacity to visualize IPC gaps, and the ability to provide immediate recommendations for improvement. These advantages allowed the NYSDOH to rapidly work with SNFs to implement solutions to unfamiliar situations, such as PPE use. The remote IPC assessment tool used during the pilot project was developed early in the pandemic, when limited knowledge of the pathogen had been elucidated and IPC guidance was developing rapidly. The tool proved to be nimble and was modified to capture evolving IPC guidance. This pilot project provided proof of principle, and remote assessments have been adapted for COVID-19 prevention and response activities across New York State and in various healthcare facility types including ACFs and hemodialysis centers. Additionally, this method has been further adapted by the CDC to address the evolving recommendations and guidance for COVID-19. Updated tools now include assessments of resident cohorting, facility testing practices, and more structured conversations on PPE optimization practices.^11^ During times when IPC activities need to be rapidly scaled up, COVIDeo may serve as a practical and successful complement to on-site assessments or telephone-only assessments.

## Supplementary material

For supplementary material accompanying this paper visit https://doi.org/10.1017/ice.2021.100. click here to view supplementary material
