# Supplementary material for: Real-time virtual infection prevention and control assessments in skilled nursing homes, New York, March 2020—A pilot project
Source: Infect Control Hosp Epidemiol. 2021 Mar 19:1–7. doi: 10.1017/ice.2021.100 (PMC8082126; doi:10.1017/ice.2021.100)
Supplement: Supplementary file 1 [file S0899823X21001008sup001.docx]

**Screening Questionnaire for NHs**

Facility Name: ______________________________ DOH Staff: ______________________ Date: _____________

Introduction:

Hi, my name is _______________________ and I’m calling from the New York Department of Health. Can you please connect me with your director of nursing or the infection preventionist of the facility?

Hi _______________ Reintroduce yourself: my name is _______________________ and I’m calling from the New York Department of Health.

Are you the best point of contact that the health department can reach 24 hours/day regarding infection control?

**Name of POC**: _________________________________________ **Phone**: __________________________

**Email address:** ______________________________

As I’m sure you’re aware, there is active COVID-19 activity in the state of New York and throughout New York City. The purpose of this call is to help assess your facility’s infection control practice in response to the Health Advisory sent out on March 13, 2020. We would like to assist your facility in assuring that all the infection control recommendations have been implemented. If your facility has had any challenges in implementing these recommendations, we would like to understand them and help mitigate the gaps. Are you able to give us 15-20 minutes of your time to understand the current situation at your facility, the infection control measures that have been implemented? Thank you, I’m going to send an email with an infection control checklist that I would like to walk through with you.

If this is not the best time, can you please let me know when I can call back?

**Screening Questions:**

1. Number of beds in the facility: ________

2. Total number of residents in the facility: ___________

3. Total number of units: __________

Specialty Units:  Vent/trach Dialysis Dementia Rehab

| **COVID-19 Question** | **Residents** | | **Staff** | |  |
| --- | --- | --- | --- | --- | --- |
| **Questions** | **Yes/No** | **#** | **Yes/No** | **#** | **Notes** |
| 3. Does your facility have any residents or staff under investigation for COVID-19 or a confirmed case? |  |  |  |  |  |

**If No**,

| **Questions:** | **Yes/No** | **#** | **Notes** |
| --- | --- | --- | --- |
| 4. Do you currently have anyone in your facility experiencing influenza-like illness (ILI)? |  |  |  |
| 5. Have any had a positive RVP? |  |  |  |

| **Questions** | **Yes/No** | **#** | **Notes** |
| --- | --- | --- | --- |
| 6. Floors/Units with COVID-19 or ILI | N/A |  |  |
| 7. Have any residents with COVID-19 or ILI been transported to a hospital? |  |  |  |
| - If yes, hospital name | N/A | N/A |  |
| 8. Have you had an increase in staff calling out? |  |  |  |
| 9. Do you know the reason for staff absenteeism? | N/A | N/A |  |

**If Yes to either question 1 or 2**

Complete the following if possible: NH/ALF checklist and virtual assessment

**COVID-19 Prevention and Control Checklist for Nursing Homes and Adult Care Facilities**

Adapted from: NYDOH Health Advisory: COVID-19 Cases in Nursing Homes and Adult Care Facilities, March 13, 2020 and

Coronavirus Disease 2019 (COVID-19) Preparedness Checklist for Nursing Homes and other Long-Term Care Settings, March 10, 2020

Key Messages:

- Any residents with influenza like illness or unexplained respiratory infections should be immediately isolated to their room.
- Residents should wear a facemask if any staff enter residents’ rooms.
- Healthcare personnel should adhere to standard, contact, and droplet precautions plus eye protection when interacting with residents experiencing respiratory illness and fevers.

Please contact your local or state health department with questions or concerns related to infection control advice, resource shortages including personal protective equipment, essential medications, and staffing related to the COVID-19 outbreak.

| **1. Facility Restrictions and Health Checks:** | | |
| --- | --- | --- |
| Implemented | Details | Not Implemented |
| ☐ | Implemented state required suspension of all visitation. | ☐ |
| ☐ | Is the facility performing active health checks including temperature monitoring for everyone entering the facility (i.e. essential visitors, HCP and staff)? | ☐ |
|  | Are staff performing health checks wearing facemasks? |  |
|  | Has the facility canceled group activities and communal dining? |  |
|  | Post signs at the entrances to the facility advising that no visitors may enter the facility |  |
| ☐ | Requiring all essential visitors to wear facemasks while in the facility and visitors should remain in residents’ room. | ☐ |
| ☐ | Are HCP and staff with symptoms or T ≥ 100.0 F sent home. HCP and staff who develop symptoms or fever while in the facility should be immediately sent home. | ☐ |

This document is a guide but does not replace clinical judgement and complements all existing infection control guidelines. Note implementation of this tool may vary for Adult Care Facilities.

| **2. Personal Protective Equipment (PPE) for Healthcare Personnel and other Staff:** | | |
| --- | --- | --- |
| Implemented | Details | Not Implemented |
| ☐ | Are HCP and other staff wearing masks throughout the facility? | ☐ |
| ☐ | Alcohol based hand sanitizers for use of hand hygiene are available inside outside every resident room, common areas, facility entrance, elevators  If hand sanitizer dispensers are not located in each resident room, is there a sink inside the resident room where staff perform hand hygiene bathroom only common area | ☐ |
| ☐ | HCP caring for residents with influenza-like illness (including those Persons Under Investigation and/or confirmed COVID-19 cases) should use the following precautions; Standard, Contact (gown and gloves), and Droplet (surgical mask with eye protection). | ☐ |
| ☐ | Necessary PPE is available immediately outside of the resident room and in other areas where resident care is provided. | ☐ |
| ☐ | Signs are posted immediately outside of resident rooms indicating appropriate IPC precautions and required PPE. | ☐ |
| ☐ | Trash disposal bins should be positioned near the exit inside/outside of resident rooms. | ☐ |
| ☐ | Ensures *EPA-registered hospital-grade disinfectants are available for frequent cleaning of high-touch surfaces and shared resident care equipment. | ☐ |
|  | Facility has a process to ensure shared non-dedicated equipment is cleaned and disinfected after each patient use according to manufacturer’s recommendations. |  |

**Notes:**

| **3. Identification and Management of Ill Residents in Facilities with Confirmed Cases of COVID-19** | | |
| --- | --- | --- |
| Implemented | Details | Not Implemented |
| ☐ | A process has been put in place to identify and manage ALL residents with symptoms of respiratory infection (e.g., cough, fever, sore throat) daily. | ☐ |
| ☐ | Residents should wear a face mask when HCP or other care providers enter their rooms, unless such is not tolerable. | ☐ |
| ☐ | Implement active monitoring of all residents on affected units once per shift. Monitoring MUST include a symptom check, vitals, lung auscultation, and pulse oximetry. | ☐ |
| ☐ | Assure all residents in affected units remain in their rooms as feasible. | ☐ |
| ☐ | Do not float staff between units. | ☐ |
| ☐ | For residents who initially test negative, re-testing should be performed immediately if they develop symptoms consistent with COVID-19. | ☐ |
| **4. Occupational Health:** | | |
| Implemented | Details | Not Implemented |
| ☐ | The facility instructs HCP and other staff to regularly monitor themselves for fever and symptoms of respiratory infection, as a part of routine practice. | ☐ |
|  | Facility should monitor HCP and staff absenteeism for increased numbers and assess the reason. | ☐ |
| **5. Communications:** | | |
| Implemented | Details | Not Implemented |
| ☐ | Communication of interfacility transfers for residents with respiratory symptoms/suspect COVID-19 notifying **EMS transfer personnel** and **receiving facilities**. Best practice would be through written and warm hand-off to the facility. | ☐ |
| ☐ | Proactively notify all resident family members about the COVID-19 activity within the facility and measures implemented. | ☐ |
| **6. Surge Capacity:** | | |
| Implemented | Details | Not Implemented |
| ☐ | Estimates should be made and frequently reassessed of the quantities of essential resident care materials and equipment (e.g., intravenous pumps and ventilators, pharmaceuticals) and personal protective equipment (e.g., masks, respirators, gowns, gloves, and hand hygiene products). | ☐ |
| ☐ | A plan has been developed to address likely supply shortages (e.g., personal protective equipment), including strategies for using normal and alternative channels for procuring needed resources. | ☐ |
| **7. Any Infection Control Challenges or Educational Requests?** | | |
|  | | |
| Footnote: * Products with EPA-approved emerging viral pathogens claims are recommended for use against COVID-19. If these are not available, products with label claims against human coronaviruses should be used according to label instructions. | | |

**Virtual IPC Visit Assessment Form**

Facility Name: ___________________________________________ Date: _________________

This “virtual visit” tool using smart phone video capabilities followed a phone call to the facility where an initial survey was conducted to evaluate the facilities current status of residents and staff with suspected or COVID positive cases. Next a step by step checklist assessment that incorporated CDC guidelines was used to assess each facility’s ability to implement COVID-19 IPC recommendations. Finally, the virtual visit was conducted where IPC advice based either on gaps identified from the checklist or seen during the virtual walk through were provided.

**Are you willing to walk us through your facility using Skype or Facetime? Y/N**

Script: Could you start outside of the building and walk us through the facility and up to a floor? This will take approximately 10 minutes.

1. **Restriction of Visitors**

Signs posted on front door
 Front doors locked

1. **Health checks for everyone entering the building**

Health check station set up near the entrance

Actively checking temperatures

Universal masking throughout the facility

1. **Hand hygiene/Alcohol dispenser**
   Entry of facility, near elevators, throughout floors **(circle all that apply)**
    Dispensers inside resident rooms
    Dispensers outside resident rooms
    Sink/s for staff to perform hand hygiene inside the resident room
2. **Signage outside resident rooms** Precaution on affected rooms
    Required PPE posted outside resident door

Signs in plastic pouches

1. **PPE storage**
    PPE located in a central location on the floor

PPE located outside of resident rooms
 Disposal bin right outside resident rooms

Disposal bin right inside resident rooms

1. **EVS**
    Ensures *EPA-registered hospital-grade disinfectants are available for frequent cleaning of high-touch

surfaces and shared resident care equipment

Can you show me the hospital-grade disinfectants that are on-site?

Additional cleaning on the floors (common areas, equipment)

1. **Additional Notes:**
